# Novel indicators to better monitor the collection and recovery of (critical) raw materials in WEEE: Focus on screens

**DOI:** 10.1016/j.resconrec.2020.104772

**Published:** 2020-06

**Authors:** Rachel Horta Arduin, Fabrice Mathieux, Jaco Huisman, Gian Andrea Blengini, Carole Charbuillet, Michelle Wagner, Cornelis Peter Baldé, Nicolas Perry

**Affiliations:** a Université de Bordeaux, ISM, UMR 5255, F-33400 Talence, France; b Arts et Métiers, Université de Bordeaux, CNRS, Bordeaux INP, I2M Bordeaux, UMR 5295, F-33405, Talence, France; c European Commission – Joint Research Centre, Sustainable Resources Directorate, Via E. Fermi 2749, 21027, Ispra, Italy; d Institut Arts et Métiers de Chambéry, I2M Bordeaux, UMR 5295, F-73375 Le Bourget du Lac, France; e United Nations University, Vice Rectorate in Europe, Sustainable Cycles Programme (SCYCLE), Platz der Vereinten Nationen 1, 53113 Bonn, Germany

**Keywords:** WEEE, Secondary raw materials, Collection rate, Recycling rate, Collection quality, Scavenging, Ag, Silver, Al, Aluminum, Au, Gold, Co, Cobalt, CRM, Critical Raw Material, CRT, Cathode-Ray Tube, Cu, Copper, EC, European Commission, EEE, Electrical and Electronic Equipment, EU, European Union, Fe, Iron, FPD, Flat Panel Display, In, Indium, LCD, Liquid-Crystal Displays, LED, Light Emitting Diode, Li, Lithium, Mg, Magnesium, Nd, Neodymium, PCB, Printed Circuit Board, Pd, Palladium, POM, Placed on the Market, PMMA, Polymethylmethacrylate, Sb, Antimony, TFT, Thin-Film-Transistor, WEEE, Waste Electrical and Electronic Equipment

## Abstract

Currently, in the European Union (EU), e-waste chain performance is assessed by technical indicators that aim to ensure system compliance with collection and recovery targets set by the WEEE Directive. This study proposes indicators to improve WEEE flow monitoring beyond the current overall weight-based approach, including complementary flows and treatment performance. A case study focused on the screen category in France is presented. In 2017, the collection rate of cathode-ray tube screens (CRT) was 68%, while for flat panel display (FPD) generated only 14% was collected. CRT screens have less precious and critical materials than FDP. Thus, elements like cobalt and gold highly concentrated in FPD, have a collection rate two to four times lower than elements such as copper (37%) which represents a high proportion in CRTs. Recycling is the main treatment in France. Nevertheless, the recycling rate per element varies significantly due to the low collection, and also the lack of technology and/or secondary raw materials market. The elements with higher recycling rates are base metals such as copper (28%), followed by precious metals like silver (23%), and gold (13%). Except for palladium, the recycling rate of the critical raw materials targeted in the study ranged from 6% (cobalt) to 0% (e.g. neodymium and indium). The results stress the need for indicators to support the development of WEEE chain from waste management to secondary (critical) raw materials suppliers.

## Highlights

• A method to track the fate of materials in e-waste flows in EU is presented.
• An extension to the current indicators to assess WEEE treatment systems is proposed.
• Recovery performance of waste screens in France is assessed per target elements.
• The type of screens collected impact the materials potentially available for recycling.
• Tracking the quality of WEEE flows can boost (critical) raw materials recovery.

## 1 Introduction

### 1.1 General background

The production of electrical and electronic equipment (EEE) is rising worldwide, alongside a decrease, mainly in industrialized societies, in the lifespan of products such as small electronic devices (Bakker et al., 2014; Ikhlayel, 2018). Consequently, the amount of electronic waste (e-waste) or waste of electrical and electronic equipment (WEEE) is rising globally, with an annual growth of approximately 5% (Ibanescu et al., 2018). In 2016, 44.7 million tons (Mt) of WEEE were generated worldwide (Baldé et al., 2017a,Baldé et al., 2017a). However, similarly to EEE production, this amount is growing at different rates in different counties (Tansel, 2017). Nowadays, there are no harmonized datasets available for sales at a global level that cover all countries in the world over more than a decade (Baldé et al., 2017a,Baldé et al., 2017a). In 2012, about 60 Mt of EEE were placed on the market (POM), among which 12.4 Mt in China, 9.1 Mt in the EU, 7.4 Mt in the USA, 3.0 Mt in India and 3.7 Mt in South America (Eurostat, 2018; Xavier et al., 2018).

Based on e-waste composition, its treatment is driven by three main benefits/reasons: economic, environmental, and public health. From an economic perspective, WEEE contains a variety of materials, including precious metals (e.g. gold, silver and palladium) and other valuable metals (copper, aluminum and iron) (Ibanescu et al., 2018). From a resource perspective, recycling of e-waste, at a certain scale, decreases the pressure on the environment due to the extraction of raw materials from mineral deposits (Laurenti et al., 2015; Meshram et al., 2019). Recycling can also contribute to the security of raw materials supply and lead to a more circular economy (Mathieux et al., 2017). Nonetheless, WEEE also contains hazardous substances that represent risks for human health and/or the environment (such as brominated flame retardants and toxic metals like mercury and lead as well as CFCs) that require specific treatment (Lixandru et al., 2017; Zhang and Xu, 2016).

### 1.2 WEEE management in Europe and current indicators

The regulation of e-waste varies significantly between countries, as well as the development level of WEEE management systems (Golev and Corder, 2017; Tansel, 2017). In the EU, WEEE management is regulated by the WEEE Directive. The first version of the WEEE Directive (Directive 2002/96/EC) came into force in 2002 parallel with the RoHS Directive (Directive 2002/95/EC) that restricted the use of certain hazardous substances in EEE products (Friege, 2012; Kumar et al., 2017).

After a few years, both Directives were revised, and the new (recast) WEEE Directive (2012/19/EU) entered into force in August 2012. The WEEE Directive aims to prevent the generation of e-waste, as well as to improve the performance of the treatment operations for the reuse, recycling and other forms of recovery (Wäger and Hischier, 2015). The WEEE Directive establishes three technical indicators to monitor WEEE systems efficiency: collection rate, recycling, preparation for reuse rate, and recovery rate (art. 3 of the WEEE Directive). The WEEE categories, according to the recast WEEE Directive, are presented in Table S1 in Supplementary Material.

The revision of the collection rate calculation, and of the targets to be achieved by the Member States are among the main changes in the WEEE Directive recast. The first WEEE Directive required Member States to collect at least 4 kg per capita of WEEE from households. From 2016 to 2018, the minimum collection rate was 45% calculated on the total weight of WEEE collected, expressed as the percentage of the overall weight of EEE placed on the market (POM) in the three preceding years. As stated in the Directive, from 2019, the Member States can report their collection rate based on the EEE POM, or in the WEEE generated approach. By 2019, the minimum collection rate should be 65% of the EEE POM, or 85% if calculated based on the WEEE generated approach (European Commission, 2012).

The WEEE generated approach considers the amount of waste leaving the stock once discarded, taking into account the lifespan of electronic equipment (Huisman et al., 2017). It includes waste collected by the official compliance schemes, as well as those captured by complementary flows. The term complementary flows refers to all e-waste flows that are not reported at a national level by the official compliance systems. A certain portion of these flows is exported, incinerated or landfilled (Huisman et al., 2017).

In order to have a common methodology for calculating WEEE generated in the EU, the European Commission (EC) published the WEEE Calculation Tool^1^ (based on Magalini et al., 2015; see Section 3.1).

So far, the POM metric is the common approach for communication and comparison of collection rates between the Member States, together with the collection of e-waste per capita. The collection rate diverges significantly between the Member States (Vidal-Legaz et al., 2018). In 2016, for example, the collection rate was 66% in Sweden and 10% in Malta (Eurostat, 2018).

Regarding the recycling and reuse rate, the WEEE Directive establishes that it should be calculated, for each WEEE category, by dividing the overall weight that enters the recycling/preparing for reuse facility by the overall weight of e-waste collected, expressed as a percentage (art. 3 of the WEEE Directive).

The current WEEE Directive indicators allow a limited overview of WEEE management since recovery performances are measured in an overall weight-based approach, without considering treatment losses and excluding flows not collected by the official schemes and captured by complementary flows (Haupt et al., 2016; Parajuly et al., 2017). Moreover, no incentives to recover specific materials, such as critical raw materials (CRM), are set by the legislation (Chancerel et al., 2015; Friege, 2012; Kumar et al., 2017; Nelen et al., 2014; Van Eygen et al., 2016).

### 1.3 Novelty and goal of the study

Information regarding WEEE flows and secondary raw material availability is useful at various levels of decision making, including for EU and national policy-makers, compliance schemes, recyclers and even designers. Nonetheless, the indicators currently in use provide neither an adequate picture of the amount of secondary resources available in e-waste, nor information about the final destination of these materials (Haupt et al., 2016).

This study, by better tracking the quality of WEEE flows, proposes an expansion of the current indicators proposed in WEEE legislation, aiming to boost (critical) raw materials recovery. In past years, other studies applied and/or proposed indicators to assess WEEE chain performance, including metrics beyond the overall weight-based approach. Some studies calculated the WEEE chain performance according to the WEEE Directive (e.g. Wen et al., 2009), and others discussed the limitations of these indicators. Huisman (2003) developed the quotes for environmentally weighted recyclability (QWERTY) approach focused on the determination of environmentally weighted recycling scores. Nelen et al. (2014) developed a set of performance indicators including economic, environmental and criticality priorities. Haupt et al. (2016) proposed that the recycling rate be calculated separately according to the type of recycling: open-loop and closed-loop.

The novelty of this study is the scope of the indicators, and the methodological approach suggested. The boundaries of collection and recycling rate indicators are enlarged in order to consider the complementary flows and treatment performances. Moreover, this study comprehensively describes the methodology used and validates the indicators in a case study. Considering the current information tracked by the official schemes, possible solutions for obtaining the necessary data to adopt the indicators are discussed. The main objectives of this study are hence to:

- Identify adequate metrics to highlight the need for improving WEEE flow tracking to monitor WEEE schemes progress;
- Present a new approach for calculating the collection and recycling rate, based on WEEE flow data, their composition and the performance of treatment processes;
- Identify the main challenges for the elements targeted in this study, to improve their recycling rates;
- Validate the new approach with a case study of the screens category in France;
- Discuss the feasibility and usefulness of such novel indicators.

The article illustrates the methodology using the example of screens (category II of WEEE Directive – see Table S1 in Supplementary Material) in France as one of the Member States transposing the WEEE Directives into national legislation, but the indicators suggested could be applied to different countries and WEEE categories. Screens were selected as a case study since they represent well how a change in technology influences the volumes and types of material available for collection and recycling (Cucchiella et al., 2015).

## 2 Materials and methods

After the assessment of the current WEEE Directive indicators and their limitations (Section 1.2), supplementary indicators are proposed to improve performance monitoring. In this section, the new indicators are expressed in equations, and the data required to perform the calculations are presented in Section 3.

The current indicators are based on the overall weight of electronic waste and focus on e-waste management. Conversely, the indicators proposed in this study intend to provide information on the WEEE chain treatment as a potential supplier of secondary raw materials. They allow the collection and recycling performance per target element to be tracked. The indicators are based on three parameters: (1) WEEE flows; (2) composition of (W)EEE; and (3) treatment performance.

An updated method is presented, including the data used to define the new input parameters (Section 3). The methodology used to map WEEE flows in France is presented in Section 3.1. The composition data used to qualify the flows, as well as the elements targeted in the study, are presented in Section 3.2. Furthermore, various treatment scenarios for screens considered in the study, as well as their performance, are detailed in Section 3.3. Section 4 summarizes the subsequent results for the screens case study in France.

**Fig. 1:**
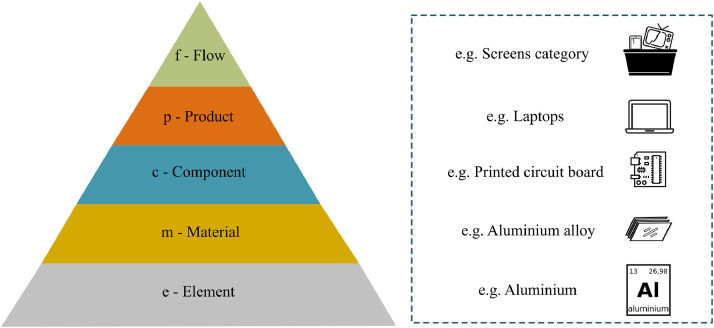
ProSUM classification system (Adapted from Huisman et al., 2017).

The indicators are based on the classification system developed in the ProSUM project for assessing (W)EEE composition (Fig. 1). The core of this system lies in the representation of waste flow (‘f’) as a regrouping of products (‘p’) that are the sum of their constituent components (‘c’), materials (‘m’) and elements (‘e’) (Huisman et al., 2017).

The collection rate based on waste generated is the basis of the indicators proposed in this work. This approach allows us to assess the performance of the official schemes, including the complementary flows, in comparison to actual waste volumes. Eqs. (1) and (2) present the collection and recycling rate considered in this study, based on the weight of waste generated per WEEE category.

(1) $$CR_{f}=\frac{\sum_{i=1}^{n}WC_{i}}{\sum_{i=1}^{n}WG_{i}}$$

(2) $$RR_{f}=\frac{\sum_{i=1}^{n}WR_{i}}{\sum_{i=1}^{n}WG_{i}}$$

where *CR_f_* is the collection rate of the WEEE category (f); *RR_f_* is the recycling rate of the WEEE category (f); *n* is the number of different UNU-Keys related to the WEEE category (UNU-Keys is a classification developed by the United Nations University to categorize different `baskets’ of WEEE products according to composition and lifespan properties (Baldé et al., 2015)); *WC* is the weight of e-waste collected by the official schemes (Deprouw et al., 2018, 2017; Monier et al., 2016, 2015, 2014, 2013); *WG* is the weight of e-waste generated (calculated based on the amount of EEE placed on the market in the preceding years, and on the corresponding product lifespan (Deprouw et al., 2018, 2017; Fangeat, 2017)); *WR* is the weight of e-waste that enters the recycling facilities (Deprouw et al., 2018, 2017; Monier et al., 2016, 2015, 2014, 2013). See Section 3.1 for further details on the screens flow tracking.

The current metrics to calculate the collection rate according to the WEEE Directive (see Section 1.2) consider a macroscopic assessment of the collection performance for all e-waste categories. The approach suggested in Eq. (1) (*CR_f_*) should be applied per WEEE category.

In order to assess the collection and recycling rate per element, data regarding the WEEE composition and treatment performance parameters are required. Eqs. (3) and (4) present the collection and recycling rate calculation per target element.

(3) $$CR_{e}=\frac{\sum_{i=1}^{n}WC_{i\;}\times \;\sum_{j=1}^{n^{\prime }}CH_{eij}\times \left(1-SG_{J}\right)}{\sum_{i=1}^{n}WG_{i\;}\times \sum_{j=1}^{n^{\prime }}CH_{eij}}$$

(4) $$RR_{e}=\frac{\sum_{i=1}^{n}WC_{i\;}\times \sum_{j=1}^{n^{\prime }}CH_{eij}\times \left(1-SG_{j}\right)\times \sum_{k=1}^{n^{\prime \prime }}SR_{ek\;}\times PR_{ek}}{\sum_{i=1}^{n}WG_{i\;}\sum_{j=1}^{n^{\prime }}CH_{eij}}$$

where *CR_e_* is the collection rate of the target element (e) in the WEEE category (f); *RR_e_* is the recycling rate of the target element (e) in the WEEE category (f); *n*′ is the number of components (c) and materials (m) present in the different products (p); *CH_e_* is the element (e) content per component (c) and materials (m) in the products (p); *SG* is the scavenging rate per component (c) (components’ scavenging level from WEEE collected by the official schemes - see Section 3.1); *n*″ is the number of treatment scenarios for all the products (p); *SR_e_* is the sorting and shredding rate of the fraction (f*) that contains the element (e); *PR_e_* is the efficiency of the final recycling operation of the target element (e).

*RR_e_* quantifies only the elements recycled into secondary raw materials with the same or similar properties (closed or semi-closed loop recycling approach). Thus, it does not capture alloying elements in secondary metals recycled in an open-loop approach (often with lesser quality and reduced functionality).

## 3 New input parameters

### 3.1 Tracking screen flows

The starting point of the study is the quantification of WEEE generated. This parameter is essential to calculate the indicators presented in Section 2. As presented by Wang (2014), different methods for quantifying e-waste generation are discussed in the literature.

We suggest using the methodology with the WEEE Calculation Tool developed by the EC to calculate WEEE generated in a given year. This approach is based on the amount of EEE POM in the preceding years, and on the corresponding product lifespan (Baldé et al., 2017a,Baldé et al., 2017). Forti et al. (2018) defined this parameter and the related mathematical equations based on the Weibull distribution. This probability function describes the discard behavior for EEE.

The pre-filled data on the quantities of EEE POM available in the tool are calculated based on the apparent consumption methodology. The apparent consumption uses available statistical data of domestic production (PRODCOM statistics) plus imports and exports (CN codes) to quantify POM (Magalini et al., 2015). The data in the tool is manually updated with recent POM data in France (2016 and 2017) from the national registers of the French Environment & Energy Management Agency (ADEME) (Deprouw et al., 2018, 2017; Fangeat, 2017).

In order to be able to calculate the lifespan per product, the tool converts the POM input data per WEEE category into POM per UNU-Keys. The UNU-Keys classification divides different types of WEEE items (about 900 products, clustered into 660 main product types, clustered in sub keys with common compositions, sizes and lifespans) into 54 categories (Baldé et al., 2015). Each category of e-waste is composed of different UNU-Keys. The screen category comprises both the cathode-ray tube (CRT) and flat panel display (FPD) screens. The UNU-keys related to the category targeted in this study (new category II - screens), as well as the amount generated in 2017 in France are presented in Table S2 in Supplementary Material.

The UNU-key 0303 comprises tablets and laptops, and a share of 7% and 93% of the total weight, respectively, for the tablets (030301) and laptops (030302) is considered based on data from the Urban Mining Platform^2^ (Huisman et al., 2017).

The Weibull distribution function then models the lifespan profile of each UNU-key according to shape and scale parameters defined for each UNU key, derived from multiple country studies (Baldé et al., 2017a,Baldé et al., 2017; Forti et al., 2018; Magalini et al., 2015).

The second step in tracking the screen flows aims at quantifying the screens collected by the official schemes, as well as the complementary flows. Data on screens collected are obtained from ADEME (Deprouw et al., 2018, 2017; Monier et al., 2016, 2015, 2014, 2013). Currently, the national registers only track the total amount of WEEE collected and treated per category and/or waste streams. Usually, there is no detailed assessment in terms of breakdown of the flow per type/category of products and, or their clustering per UNU-key.

In order to convert the data of WEEE collected per category in UNU-keys, data provided by the ProSUM project and Ecologic, one of the compliance schemes in France, is used. For the EU 28+2, the ProSUM project determined an average share of UNU-keys collected per Member States (Huisman et al., 2017). According to data provided by Ecologic, until 2016, screens collected by the official schemes were mainly CRT (90%). In 2017 it changed slightly, and FPD represented approximately 15%. The share of UNU-keys considered in WEEE collected in France is presented in Table S3 in Supplementary Material.

WEEE not collected by the official schemes ends up in other non-documented routes (complementary flows), including equipment reused, e-waste disposed in the municipal waste stream, as well as undocumented exports either as (illegal) waste or as reusable items (Bigum et al., 2013; Huisman et al., 2015; Vidal-Legaz et al., 2018). Another relevant source of non-documented flows from the official schemes is component scavenging (Huisman et al., 2017). Scavenging is a consequence of plundering in the e-waste collection points, and also of components removed by the consumers before disposing of the e-waste. Based on a study performed by EERA (Magalini and Huisman, 2018), it can be substantiated that key valuable components are scavenged, for example 30% of cables from the reported flow of screen devices. The other screen components and corresponding percentages considered to be scavenged are presented in Table S4 in Supplementary Material.

### 3.2 Screen compositions

To track the target elements in e-waste flows, data about the composition of products and waste flows is essential. The composition of screens depends on producers, the functionalities of the device, and changes in technologies. Nowadays, producers do not report EEE composition, and the compliance schemes usually do not sample e-waste composition on a regular basis.

Data from the ProSUM project is used for the UNU-keys composition of the screen category (see Table S5 in Supplementary Material). The advantage of this classification is that it allows a uniform description of all composition parameters, like the share of products (e.g. per UNU key) to the waste flow (p-f) and the metal content in a particular alloy (e-m) used in specific components (m-e). This information is necessary to determine the recoverability of elements based on the technological processes available. Often, WEEE composition data is provided as the content of the elements in flows or products (e.g. the quantity of antimony or gold in tablets). However, the recoverability of the elements is influenced by their content in materials and components. For example, antimony can be recovered efficiently in processes based on complex lead/copper/nickel metallurgy (>80%), but antimony in plastics and CRT glass is generally not recycled (Chancerel et al., 2015; Hagelüken, 2006).

After a first analysis of screen compositions with data available in the Urban Mine Platform, we selected some target elements for this study. As previously mentioned, e-waste treatment is driven by economic, environmental, and public health considerations. Thus, instead of selecting only elements that are present in significant quantities by weight (the main focus of WEEE chain to comply with the weight-based targets), we also selected elements present in smaller quantities but with high intrinsic value (market price) and/or included in the 2017 CRMs list for the EU. The following 11 elements were selected: (1) critical raw materials: antimony (Sb), cobalt (Co), indium (In), magnesium (Mg), neodymium (Nd) and palladium (Pd); (2) non-critical raw materials: aluminum (Al), gold (Au), silver (Ag), copper (Cu) and lithium (Li). Lithium was considered in this study even if its intrinsic value is rather average (3 times higher than copper but 2000 times lower than gold), and it is not considered a CRM in the last list because its economic importance is slightly below the limit (≥2.8). However, lithium supply risk is above the limit considered in the criticality zone (≥1), and with its rapidly growing consumption, the economic importance may change in the future criticality assessment.

The targeted elements are present in different components of CRT and FPD screens. Aluminum and magnesium are present in significant quantities in different types of alloys in screen casings. Among others, PCBs contain precious metals (Ag, Au and Pd), as well as base metals like aluminum and copper. Copper is also present in cables and CRT screen motors. Antimony is mostly found in plastic components (applied as a synergist for brominated flame retardants), and CRT glass. Lithium and cobalt are mainly present in batteries in tablets and laptops. Indium and neodymium are mainly present in FPD, respectively, in thin-film-transistor (TFT) in liquid-crystal displays (LCD) and magnets in drives.

### 3.3 Screen treatments

Between the e-waste collection by the official schemes and the actual production of secondary raw materials, there is a complex chain of processes driven by economic and environmental interests (Mathieux et al., 2008; Valero Navazo et al., 2013). This chain includes several stakeholders in charge of transport, storage, clean-up, shredding and sorting (Tesfaye et al., 2017).

In order to calculate the recycling rate of the official schemes per target element, various treatment scenarios are defined based on literature and expert judgment (researchers, compliances schemes, and recyclers) of the current practices. The treatment scenarios are described below and summarized in Table S6 in Supplementary Material.

#### 3.3.1 Televisions and monitors

Due to differences in technologies and cleaning requirements, the screens are first sorted into CRT and FPD. CRT screens include both televisions and monitors, and their treatment consists of casing dismantling, removal of the electron gun, pressurization of the CRT and, following the EU WEEE Directive, removal of the CRT and PCBs. The remaining electronics undergo shredding and separation into fractions that are sent to further recovery processes (Huisman et al., 2008; Monier et al., 2013).

FPD screens comprise a wider range of equipment types and include different types of technologies: LCD, light-emitting diode (LED) and plasma. Nowadays, in France, the FPD waste stream is mostly comprised of mercury backlights containing LCD's (Froelich and Sulpice, 2016). Mercury-free LED screens are the natural evolution of LCD screens and since they were put on the market later on, it is expected that the volume in screen waste stream will increase in the following years (Cucchiella et al., 2015).

The first step of FPD treatment is manual sorting, according to different technologies. Then, treatment is followed by dismantling (manual or mechanical) and removal of high-value components and/or hazardous components required by the EU WEEE Directive (e.g. PCBs and backlighting lamps that contain mercury). The remaining parts are forwarded to a shredder and further separation of fractions. Recently, automatic LCD recycling processes have started to be implemented in Europe (Horta Arduin et al., 2019).

#### 3.3.2 Tablets and laptops

The treatment scenarios considered are based on interviews with recyclers in Europe and identified as representative scenarios for the EU (Tecchio et al., 2018). According to the WEEE Directive, the batteries of tablets and laptops should be removed before e-waste treatment, but possibly some tablets are directly shredded mixed with other WEEE. Laptops can follow two main processing routes after battery and display panel removal: a first one based on the shredding and sorting of fractions; and a second one including a medium-depth manual dismantling of components before shredding and mechanical sorting. The manual dismantling of tablets and laptops are the most effective in terms of materials recovery. However, its use is limited due to high labor costs.

#### 3.3.3 Pre-processing and recycling

As can be noticed from the treatment scenario description, dismantling is typically followed by a size reduction step (Işıldar et al., 2018). After the waste is shredded, it is separated into different fractions (f*) by a variety of sorting techniques (Bigum et al., 2012; De Meester et al., 2019).

Due to technological limitations, output fractions of sorting processes are not pure (Horta Arduin et al., 2019). For this reason, a transfer coefficient matrix is used to determine the real quantity of elements (e) in the sorting fractions (f*) (see sorting and shredding rate in Eq. (4)). The transfer coefficient matrix used is presented in Table S7 in Supplementary Material. These values can significantly change between different companies according to the technologies used, as well as the dismantling and shredding processes before mechanical sorting.

Lastly, the efficiency of final operations that recover secondary raw materials from the sorted components and fractions (gate-to-gate approach) is also considered. This performance is commonly called “recycling rate” in the literature, but to distinguish it from the recycling rate indicators discussed in this study, it is defined here as the “efficiency of the final recycling operation” (see Eq. (4)). The efficiencies considered in this study are presented in Table S8 in Supplementary Material.

## 4 Results

### 4.1 Collection rate

**Fig. 2:**
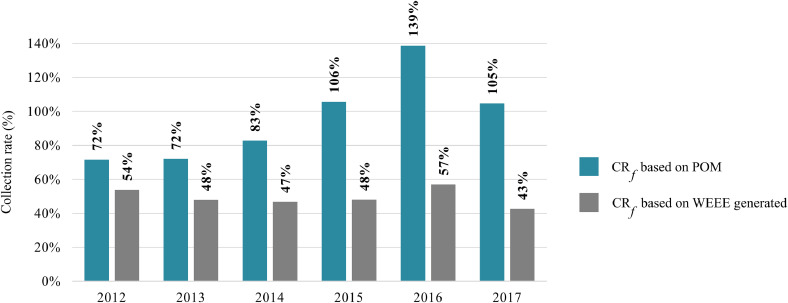
Comparison of different approaches for calculating WEEE category II collection rate (CR_f_).

Fig. 2 presents the collection rate for the screen category (CR_f_) in France from 2012 to 2017, based on the POM approach and WEEE generated (Eq. (1)). As previously discussed, the WEEE Directive does not set collection targets per WEEE category, but on the totals. Nonetheless, to monitor what is being collected by the official schemes, it is necessary to have a closer view per WEEE category.

The official schemes in France comply largely with the targets based on the POM approach. This high performance is due to the difference in the type (and overall weight) of e-waste collected by the official schemes and EEE placed on the market. The official schemes collect mostly CRT screens and since 2008 only FPD screens, much lighter, have been placed on the market. In contrast, when considering the lifetime of the different devices, the result is drastically lower. The waste generated approach shows the need to improve collection and to adopt indicators that can better monitor the performance of the official schemes.

**Fig. 3:**
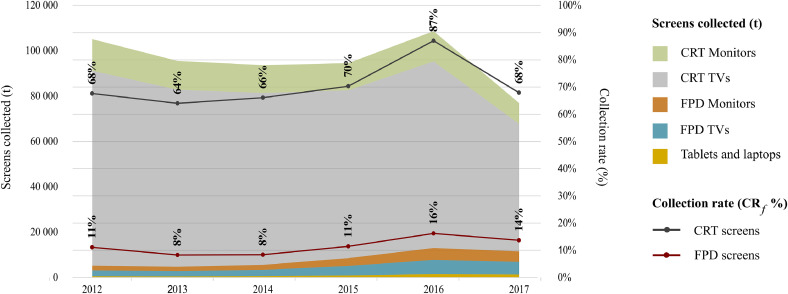
Collection performance per type of WEEE screens.

Fig. 3 presents the weight of screens collected per UNU-Keys (t), as well as the collection rate based on the waste generated approach (CR_f_) per type of screen (FPD and CRT). CRT screens have a higher collection rate in comparison to FPD screens. The high collection of CRT screens reflects changes in technology and the subsequent replacement by FPDs. The number of FPD devices in the reported collection flows reaching recycling facilities is still limited, and significantly below the share of the devices in the WEEE generated. The low collection of FPDs is influenced by the difficulties in capturing monitors, TVs, tablets and laptops due to scavenging of products, and reuse outside the official flows (including exports).

In terms of weight, the amount of FPD collected in 2017 (11,546 t) is two times higher than the collection in 2012 (5,258 t). FPD collection should increase in the following years, and over time, CRT amounts will go down to zero (Huisman et al., 2008).

FDP screens represent different types of equipment that also have different collection performances. Changes in the type of screens collected will impact collection rate results, as well as the type of materials recovered due to differences in screen compositions.

### 4.2 Collection rate per target element

**Fig. A1:**
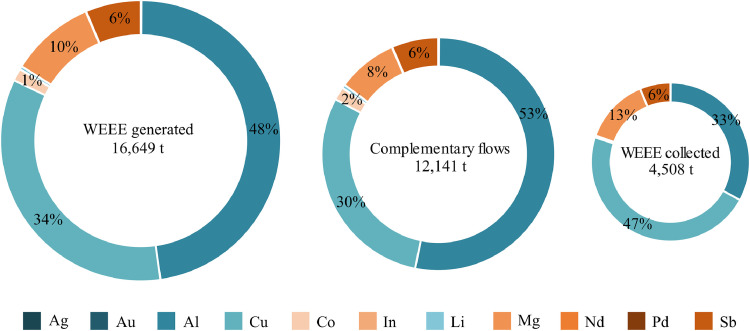
WEEE category II in France: total amount and target elements’ share (2017).

The total amount and the share of target elements in WEEE screens in France in 2017 are presented in Fig. A1 in Appendix A. From a weight-based perspective, aluminum and copper account for around 80% of the elements targeted in the study.

Silver, gold, neodymium and palladium represent only about 0.1% of the overall weight of WEEE generated and collected by the official schemes (about 22 t and 4 t in 2017, respectively). Nevertheless, precious metal recycling is the main economic driving force for WEEE recycling (> 95%) (Charles et al., 2017; Ueberschaar et al., 2017). The concentration of copper, gold, silver and palladium in the e-waste stream is significantly higher compared to primary ore grades in conventional mining operations (Kumar et al., 2017).

**Fig. 4:**
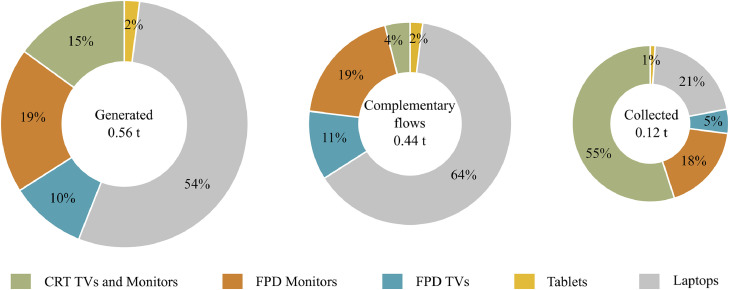
Total weight and share of palladium per UNU-key in WEEE category II in France (2017).

Fig. 4 presents the palladium content in WEEE generated per UNU-keys in France in 2017, and the subsequent share in WEEE collected by the official schemes and complementary flows. Besides being a precious metal, palladium is also in the list of critical raw materials for the EU.

Palladium and other precious and/or critical materials (e.g. Au, Ag, In and Nd) are mainly present in FPD screens (> 75% of screens generated content). As can be noticed in Fig. 4, more than 50% of the palladium collected nowadays is from PCBs of CRT devices. Due to the low collection of FPD, most of the palladium, as well as other precious and/or critical materials, are not collected by the official schemes and consequently, are not yet available in reported recycling.

**Fig. A2:**
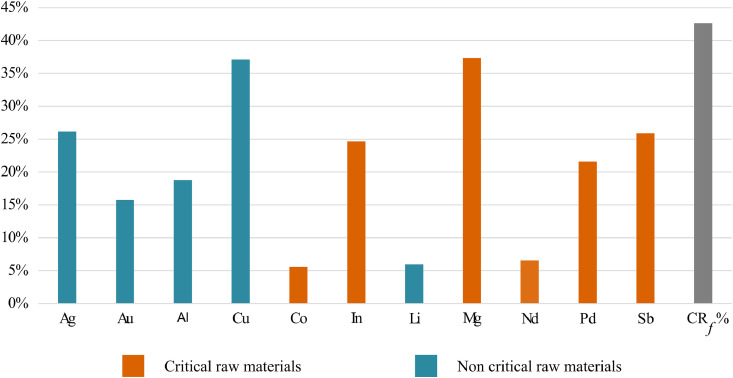
WEEE category II in France: collection rate per target element (2017).

The collection rate per element (CR_e_) in 2017 is presented in Fig. A2 in Appendix A, applying Eq. (3). All targeted elements have lower performance than the collection rate calculated based on the overall weight of screens (CR_f_) (43% as presented in Fig. 2). The differences in collection rate performances per targeted element (from 7% up to 38%) reinforce the need for more detailed monitoring. Besides the gap between the overall quantities of WEEE generated and collected, the share of the complementary flows and the quality of what is being collected by the official schemes should also be monitored.

The elements with a higher collection rate (CR_e_) are those significantly present in CRT screens (Cu and Mg, both with 37%). Even if CRT devices contain more than 50% of the Mg and Cu generated, as shown in Fig. 3, CRT's do not have a 100% collection rate. Consequently, in the case of copper, only 53% of the copper generated in CRT devices is collected by the official schemes, the remaining travels with complementary product flows and components scavenging. This, combined with the low collection of FPD devices by the official schemes (only 13% of the copper generated by FPD is collected), explains the limited resulting collection rate of the element.

The metals with medium collection performance (e.g. Al and In) are prevalent in the FPD TVs and monitors that have a growing collection rate. Lastly, the elements with lower collection rates (e.g. Co, Li and Nd with less than 10%) are significantly present in laptops and tablets whose collection rate by the official schemes is still modest (less than 10% of FPD screens collected in 2017).

**Fig. A3:**
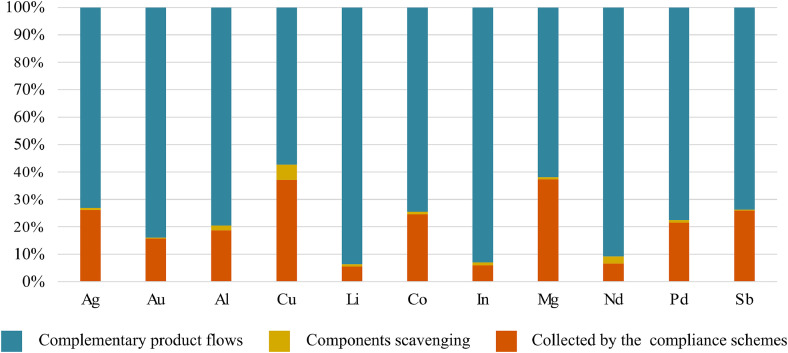
WEEE category II in France: target element generated (2017).

Scavenging of higher-valued components (e.g. PCB, drives) from the official collection channels (c-f) reduces the value of reported material content (Huisman et al., 2017). In 2017, scavenging accounted for up to 6% of the amount diverted from official schemes. The impact of component scavenging per target element is presented in Fig. A3 in Appendix A.

**Fig. 5:**
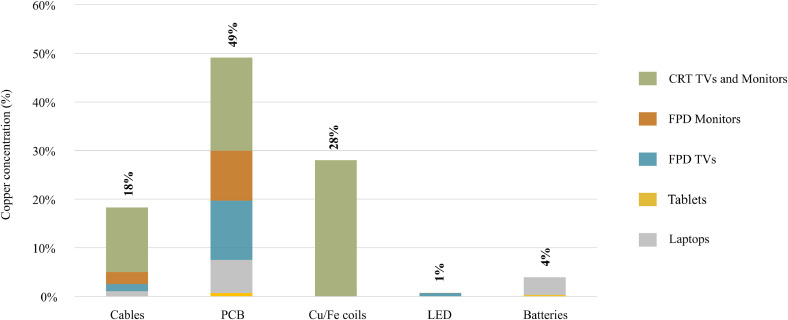
Distribution of copper per component (e-c) per UNU-key (c-p) in WEEE category II (e-f) generated (2017).

Precious metals like gold and palladium are mostly concentrated in PCB in FPD screens (above 50%). Based on EERA data (see Section 3.1), it is considered that 5% of PCBs collected are scavenged. As previously mentioned, most of the FPDs are diverted into complementary flows, consequently, the impact of components scavenging seems negligible when compared with the total generation, but it is significant for the volume collected. Copper is more affected by component scavenging, since it is present in PCB, but also in other components of different types of screens that have similar or higher scavenging, like coils (copper wire) and cables (Fig. 5).

### 4.3 Recycling rate

From 2012 to 2015, the weight-based recycling rate target for screens (former categories 3a and 4a) according to the WEEE Directive approach was 65%. From 2015, the target included both recycling and reuse and increased to 70% (European Commission, 2012). The screen recycling rate in France complies with the WEEE Directive target. The performance has been quite stable in recent years – between 82% and 85%. In this study, the recycling rate of the overall weight of screens (RR_f_) is considered as the ratio of materials sent to recycling facilities, divided by the waste generated (Eq. (2)). This approach complies with the goal of the study to understand and quantify the flows of WEEE screens.

The recycling rate results based on the WEEE generated approach (RR_f_), even without considering the sorting and recovery losses, are significantly different from the WEEE Directive approach. This difference is due to the change in the indicator scope: instead of considering only the WEEE collected by the official schemes, RR_f_ considers the WEEE generated. Thus, in 2017, 43% of WEEE generated was collected by the official schemes, and 36% of the e-waste was sent to recycling facilities – in contrast to the 82% recycling rate reported by the compliance schemes. Although the waste management hierarchy indicates waste reduction and reuse as the preferred options (Manfredi and Goralczyk, 2013), recycling is the main treatment in France. Reuse is still modest, and not well documented by the official schemes.

### 4.4 Recycling rate per target element

**Fig. 6:**
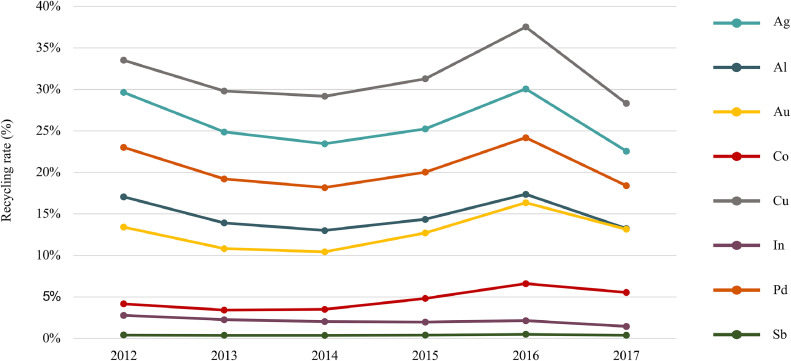
Recycling rate of target elements (RR_e_) in WEEE category II.

Fig. 6 presents the recycling rate, from 2012 to 2017, (in %) per target element recycled calculated in accordance with Eq. (4). The results consider only the elements recycled by the official schemes. In 2017, 16,718 t of target elements were generated in the screen category in France. The official schemes collected 4,521 t, and 2,707 t were recycled. Table 1 presents the amount (in tons) recycled per target element in 2017.

**Table 1:** Target elements recycled from screens in France (2017).

Target element	Target element	Amount recycled in 2017 (tons)
Non-critical raw materials	Ag	2.83
	Au	0.24
	Al	1046.51
	Cu	2,169.96
	Li	0
Critical raw materials	Co	5.98
	In	0.06
	Mg	0
	Nd	0
	Pd	0.11
	Sb	5.53
Total		2,707.19

It is assumed, based on literature and discussions with recyclers that no industrial-scale process is currently available to recover neodymium, magnesium, and lithium from batteries – hence their recycling rate is equal to zero and is not presented in the graph. Magnesium is currently recycled as part of the aluminum value stream in alloys (Mathieux et al., 2017). As mentioned in Section 2, the scope of the indicators does not include recycling in mixed alloys.

In terms of weight, aluminum and copper accounted for 99% of target elements recycled. Copper is the target element with the highest recycling rate (28% in 2017).

When comparing the differences in recycling performance from 2012 to 2017, besides the impact of the overall weight collected by the official schemes, the influence of the type of screens collected over time can be identified. Even if the total weight of screens collected in 2017 decreased in comparison to previous years, (Fig. 3), the amount of gold recycled increased - driven by the increase of FDPs collected. The same effect can be observed for elements like aluminum and palladium, even despite a decrease in the overall weight of screens collected. Nonetheless, their recycling rate has decreased due to the growth of FDPs generated in contrast to their modest collection.

Taking a closer look at cobalt, mainly present in batteries, the quantity generated from 2012 to 2017 has more than doubled. Tablets and laptops still have a modest collection, but that collection has significantly increased in the last 5 years (from 558 tons in 2012 to 1,255 t in 2017). Except for palladium, the CRMs targeted in this study have a lower recycling rate in comparison to non-critical materials.

Part of the equipment and components diverted from the official schemes is potentially recycled and reused in unofficial flows. In addition to not contributing to official targets, some of them are recycled/reused outside the EU and this results in economic and material losses for the EU. Assuming that all components scavenged from screens in France in 2017 were sent to recycling, it is estimated that around 450 t of target elements were recycled (mostly Cu and Al, but also of elements such as Au and Pd that have high intrinsic value).

## 5 Discussion

WEEE officially reported as collected by the official schemes (based on POM approach) are usually efficiently recycled in terms of overall weight. However, as illustrated by the case study, when taking a closer look at the collection and recycling efficiency of some target elements present in small quantities, amongst which are the CRMs, the results are not that impressive.

We identified that the modest results are related to four main difficulties in improving performance rates: (1) low collection by the official schemes, especially of FPD screens; (2) difficulties in manual or mechanical pre-processing; (3) absence of recycling processes at an industrial scale; (4) low economic incentives compared to recycling costs.

Manual sorting of high-value components (e.g. PCBs and drives) increases resource efficiency (in terms of the quantity and quality of recoverable materials) but entails higher labor costs (Ardente et al., 2014; Tecchio et al., 2018). The lack of efficient pre-processing mechanical technologies also limits recycling. Design plays an essential role if the recycling yields can be improved with easier dismantling and sorting of components (Ardente et al., 2014). Magnets in drives, for example, are often embedded and glued in place in the products making their extraction and recycling difficult (Lixandru et al., 2017).

Moreover, for some elements, the costs (economic and/or environmental) of recycling them as a pure element may be higher than raw material production. Seeing the limits of recycling all elements present in WEEE, a metric to consider open-loop recycling should be analyzed in a future study.

Low recycling rates of CRMs are, among others, related to the fact that processes to recover some CRMs are only found in pilot plants (Peeters et al., 2018; Zimmermann and Gößling-Reisemann, 2013). Several potential recycling processes for neodymium available in nickel-plated neodymium magnets (NdFeB) and indium in TFT panels have been described in the literature, but none of them has been developed commercially due to low productivity and high costs (Padhan et al., 2017; Ylä-Mella and Pongrácz, 2016). The increase of FPD share in screens collected may contribute to reach the critical mass necessary to make the recovery process of certain metals like indium economically viable (Ardente et al., 2014).

High recycling costs and low economic and regulatory incentives tend to discourage the recycling of certain materials. Besides neodymium and indium previously described, lithium present in batteries and Al-Li alloys is also generally not considered for recycling because virgin material is relatively low-priced (Meshram et al., 2019).

Overall, improving the recycling of CRMs from WEEE could decrease EU demand for raw materials and eventually even reduce their criticality in some cases (Blengini et al., 2017). Losses of special metals (including critical raw materials) are actually flagged by the Indicator 17 concerning WEEE management of the Raw Material Scoreboard 2018 published by the European Commission (Vidal-Legaz et al., 2018). Hence, there is a real interest for decision-makers, including business and policy-makers, to run the indicators presented in this paper. The assessment of economic viability and the potential environmental benefits of the recycling strategies for CRMs could stimulate investment in industrial-scale processes. Nevertheless, information about the content of CRMs in (W)EEE is crucial for viability studies (Peeters et al., 2018). The recent approval of eco-design requirements for servers and data storage products (European Commission, 2018) making the indication of cobalt content in batteries and neodymium in the hard-drives compulsory, can be seen as an essential initiative taken by policy-makers towards data availability and transparency to support tracking CRMs in (W)EEE.

The feasibility of compliance schemes and policy-makers to run the indicators suggested in this work regularly depends on the availability of data to calculate them. While WEEE category collection and recycling rate (*CR_f_* and *RR_f_*) could be adopted in the short term, the collection and recycling rate per target element (*CR_e_* and *RR_e_*) requires strategies to solve problems of data availability. A way to increase feasibility is to reduce the scope of the indicators to only a few selected (critical) raw materials, as also shown in Section 4.

Since *CR_f_* and *RR_f_* system boundaries consider the waste generated, the WEEE Calculation Tool should be adopted by the Member States. In France, the compliance schemes perform annual characterization campaigns to quantify the amount of WEEE collected per category, as well as the average composition per waste stream. An assessment of the share of equipment (e.g. UNU-keys) in screens collected, as well as of the components scavenged could be included in the annual characterization. As discussed by Baxter et al. (2016), future regulations should include flow monitoring via sampling and measurement. That would allow the changes in the type of equipment collected by the official schemes to be monitored, and would quantify the materials potentially available for recycling.

Regarding (W)EEE composition, collaboration between producers and recyclers to find a cost-effective and efficient way of producing and sharing product and component data would support data gathering (Downes et al., 2017). However, this is not an issue that can be solved in the short term. A short/medium-term solution is to use ProSUM data. Nevertheless, this is not a solution for the long-term because the composition of the electronic device changes rapidly with advances in technology.

## 6 Conclusions

This work proposes an extension of the current indicators to assess WEEE schemes performance. Our method is based on three input parameters: e-waste flow tracking; WEEE composition, and efficiency of recycling processes. The scope of the new indicators includes the parallel flows which divert secondary resources from the official schemes.

The results of the case study confirm that monitoring the performance per target element in comparison to overall weight indicators allows an overview of the volume and the grade of potential secondary resources. It also allows us to identify the main challenges to improving material recovery, aiming for a circular economy.

In the case of screens, we observed a significant difference in the collection performance of CRT and FPD screens. In 2017, FDP represented 47% of WEEE generated. However, CRTs represent 85% of the screens collected by the official schemes. Consequently, the collection and recycling rate of elements mainly present in FDPs, and more specifically in tablets and laptops’, is three to four times lower than the elements present in CRT devices. Thus, it is important to have indicators to monitor the quality of e-waste collected and recycled by the official schemes.

It is important to remark that the feasibility of the indicators presented in this work relies mainly on gathering data related to the three input parameters. Besides the challenges related to gathering data, it may be difficult for compliance schemes and policy-makers to continuously monitor and interpret results for many elements or materials. The development of a single score indicator calculated by weighting and summing elemental indicators could be a solution. However, it should be ensured that important data on the elements' performance would not be lost. Alternatively, national and EU policy-makers could define a limited number of elements to track based on criticality, economic and/or environmental aspects.

Together with previous studies published in the literature (Ardente et al., 2014; Haupt et al., 2016; Huisman, 2003; Nelen et al., 2014; Parajuly et al., 2017; Tansel, 2017; Van Eygen et al., 2016), this work could support the development of indicators evaluating different environmental, economic and criticality priorities related to raw materials. Future policies could adopt indicators beyond the weight-based approach and better practices in WEEE management in order to improve the e-waste tracking and recovery of (critical) raw materials. Doing so, more targeted WEEE management activities have the potential to extend their scope from waste and hazardous substances management to enhance the supply of quality secondary (critical) raw materials.

## Disclaimer

The views expressed in the article are personal and do not necessarily reflect an official position of the European Commission.

## Declaration of Competing Interest

The authors declare that they have no known competing financial interests or personal relationships that could have appeared to influence the work reported in this paper.

## Appendix A

Novel indicators to better monitor the collection and recovery of (critical) raw materials in WEEE: focus on screens

Fig. A1, A2, A3.

## Appendix B Supplementary materials

Image, application 1

## Footnotes

1 WEEE Calculation Tool is available in: http://ec.europa.eu/environment/waste/weee/data_en.htm.

2 Urban Mining Platform is available in: www.urbanmineplatform.eu.
