# Supplementary material for: Novel indicators to better monitor the collection and recovery of (critical) raw materials in WEEE: Focus on screens
Source: Resour Conserv Recycl. 2020 Jun;157:104772. doi: 10.1016/j.resconrec.2020.104772 (PMC7224517; doi:10.1016/j.resconrec.2020.104772)
Supplement: Supplementary file 1 [file mmc1.docx]

**Supplementary Material**

Novel indicators to better monitor the collection and recovery of (critical) raw materials in WEEE: focus on screens

**Summary**

This supplementary material contains the following data: (I) e-waste categories according to WEEE Directive recast; (II) screens generated per UNU-Keys in France based on WEEE Calculation Tool; (III) share of UNU-Keys in screens collected in France; (IV) percentage of components scavenged from screens; (V) screens composition based on data from the ProSUM Project ; (VI) a summary of the treatment scenarios considered in the case study; (VII) transfer coefficient matrix considered to account for shredding and sorting performance; and (VIII) the efficiency rate of the final recycling operation.

Table S1. E-waste categories according to WEEE Directive.

| **WEEE categories**  **From August 12, 2012 to August 14, 2018** | **WEEE categories**  **From August 15, 2018** |
| --- | --- |
| 1. Large household appliances | 1. Temperature exchange equipment |
| 1. Small household appliances | 1. Screens, monitors, and equipment containing screens having a surface greater than 100 cm^2^ |
| 1. IT Equipment and telecommunications | 1. Lamps |
| 1. Consumer equipment | 1. Large equipment (any external dimension more than 50 cm) |
| 1. Lighting equipment | 1. Small equipment (no external dimension more than 50 cm) |
| 1. Electrical and electronic tools | 1. Small IT and telecommunication equipment (no external dimension more than 50 cm) |
| 1. Toys, leisure and sports |  |
| 1. Medical devices |  |
| 1. Monitoring instruments and control |  |
| 1. Automatic dispensers |  |

Source: Adapted from European Commission, 2012

Tables S2. WEEE generated per UNU-keys in screens generated in France based on WEEE Calculation Tool (2017).

| **UNU-keys** | **Description** | **Waste generated** | | |
| --- | --- | --- | --- | --- |
|  |  | **t** | **%** | |
| 0303 | Laptops and tablets | 15 291 | | 8 |
| 0308 | Cathode Ray Tube Monitors | 37 929 | | 21 |
| 0309 | Flat Display Panel Monitors | 22 397 | | 12 |
| 0407 | Cathode Ray Tube TVs | 58 391 | | 32 |
| 0408 | Flat Display Panel TVs | 46 614 | | 26 |

Table S3.  Share of UNU-keys in screens collected in France by the official schemes (p-f).

| **UNU-keys** | **2012** | **2013** | **2014** | **2015** | **2016** | **2017** |
| --- | --- | --- | --- | --- | --- | --- |
|  |  |  |  |  |  |  |
| 030301  Tablets | 0,04% | 0,04% | 0,05% | 0,07% | 0,09% | 0,12% |
| 030302  Laptops | 0,52% | 0,52% | 0,63% | 0,94% | 1,25% | 1,56% |
| 0308  CRT Monitors | 13,25% | 13,25% | 13,11% | 12,69% | 12,28% | 11,86% |
| 0309  FPD Monitors | 2,00% | 2,00% | 2,41% | 3,62% | 4,81% | 6,01% |
| 0407  CRT TVs | 81,75% | 81,75% | 80,89% | 78,28% | 75,72% | 73,14% |
| 0408  FPD TVs | 2,43% | 2,43% | 2,92% | 4,40% | 5,84% | 7,30% |

Source: Ecologic and ProSUM

Table S4. Scavenging of screens components and materials (c-f, m-f)

| **Components / Materials** | **Scavenging level** |
| --- | --- |
|  |  |
| Cables | 30% |
| Drives | 32% |
| Printed circuit boards | 5% |
| Copper/iron coils and motors | 8% |
| Aluminum alloys | 15% |
| Copper alloys | 15% |
| Magnesium alloys | 15% |
| Batteries | 15% |

Source: Magalini and Huisman, 2018

Table S5. Screens composition (mg/kg UNU-Keys) (e-c, e-m)

| **UNU keys** | **Components / Materials** | **Ag** | **Al** | **Au** | **Co** | **Cu** | **Hg** | **In** | **Li** | **Mg** | **Nd** | **Pd** | **Sb** |
| --- | --- | --- | --- | --- | --- | --- | --- | --- | --- | --- | --- | --- | --- |
| 030301  Tablets | Cables | 0,00 | 309,79 | 0,00 | 0,00 | 3 029,04 | 0,00 | 0,00 | 0,00 | 0,00 | 0,00 | 0,00 | 0,00 |
|  | Drives | 0,00 | 0,00 | 0,00 | 0,00 | 0,00 | 0,00 | 0,00 | 0,00 | 0,00 | 54,02 | 0,00 | 0,00 |
|  | PCB | 96,38 | 2 513,11 | 26,41 | 46,07 | 35 424,50 | 0,00 | 0,00 | 0,00 | 154,88 | 3,91 | 0,58 | 0,00 |
|  | Cu/Fe coils, motors | 0,00 | 0,00 | 0,00 | 0,00 | 0,00 | 0,00 | 0,00 | 0,00 | 0,00 | 0,00 | 0,00 | 0,00 |
|  | Display LCD | 174,13 | 5 724,62 | 44,66 | 0,00 | 2,97 | 0,23 | 34,40 | 0,00 | 533,15 | 0,00 | 7,88 | 0,00 |
|  | Display CRT | 0,00 | 0,00 | 0,00 | 0,00 | 0,00 | 0,00 | 0,00 | 0,00 | 0,00 | 0,00 | 0,00 | 0,00 |
|  | Display TFT | 0,00 | 0,00 |  |  | 0,00 | 0,00 | 0,00 | 0,00 | 0,00 | 0,00 | 0,00 | 0,00 |
|  | Al alloys | 0,00 | 175 108,07 | 0,00 | 0,00 | 0,00 | 0,00 | 0,00 | 0,00 | 4 477,45 | 0,00 | 0,00 | 0,00 |
|  | Cu alloys | 0,00 | 0,00 | 0,00 | 0,00 | 0,00 | 0,00 | 0,00 | 0,00 | 0,00 | 0,00 | 0,00 | 0,00 |
|  | Mg alloys | 0,00 | 8 281,73 | 0,00 | 0,00 | 3,01 | 0,00 | 0,00 | 0,00 | 91 149,22 | 0,00 | 0,00 | 0,00 |
|  | Background lighting CFL | 0,00 | 0,00 | 0,00 | 0,00 | 0,00 | 0,00 | 0,00 | 0,00 | 0,00 | 0,00 | 0,00 | 0,00 |
|  | LED | 0,00 | 0,00 | 0,00 | 0,00 | 0,00 | 0,00 | 0,00 | 0,00 | 0,00 | 0,00 | 0,00 | 0,00 |
| 030302  Laptops | Cables | 0,18 | 11,07 | 0,05 | 0,00 | 4 156,58 | 0,00 | 0,00 | 0,00 | 0,00 | 0,00 | 0,01 | 0,00 |
|  | Drives | 0,00 | 0,00 | 0,00 | 15,34 | 0,00 | 0,00 | 0,00 | 0,00 | 0,00 | 505,61 | 0,00 | 0,00 |
|  | PCB | 120,26 | 3 098,41 | 53,80 | 1,67 | 27 472,38 | 0,00 | 6,03 | 0,52 | 63,69 | 9,24 | 14,49 | 317,22 |
|  | Cu/Fe coils, motors | 0,00 | 0,00 | 0,00 | 0,00 | 0,00 | 0,00 | 0,00 | 0,00 | 0,00 | 0,00 | 0,00 | 0,00 |
|  | Display LCD | 151,61 | 4 984,26 | 38,89 | 0,00 | 2,58 | 0,20 | 29,95 | 0,00 | 464,19 | 0,00 | 6,86 | 0,00 |
|  | Display CRT | 0,00 | 0,00 | 0,00 | 0,00 | 0,00 | 0,00 | 0,00 | 0,00 | 0,00 | 0,00 | 0,00 | 0,00 |
|  | Display TFT | 0,00 | 0,00 | 0,00 | 0,00 | 0,00 | 0,00 | 0,00 | 0,00 | 0,00 | 0,00 | 0,00 | 0,00 |
|  | Al alloys | 0,00 | 132 182,77 | 0,00 | 0,00 | 0,00 | 0,00 | 0,00 | 0,00 | 3 379,87 | 0,00 | 0,00 | 0,00 |
|  | Cu alloys | 0,00 | 0,00 | 0,00 | 0,00 | 0,00 | 0,00 | 0,00 | 0,00 | 0,00 | 0,00 | 0,00 | 0,00 |
|  | Mg alloys | 0,00 | 3 727,78 | 0,00 | 0,00 | 1,36 | 0,00 | 0,00 | 0,00 | 41 028,15 | 0,00 | 0,00 | 0,00 |
|  | Background lighting CFL | 0,00 | 0,00 | 0,00 | 0,00 | 0,00 | 0,39 | 0,00 | 0,00 | 0,00 | 0,00 | 0,00 | 0,00 |
|  | LED | 0,00 | 0,00 | 0,00 | 0,00 | 0,00 | 0,00 | 0,00 | 0,00 | 0,00 | 0,00 | 0,00 | 0,00 |
| 0308  CRT Monitors | Cables | 0,00 | 1 273,68 | 0,00 | 0,00 | 6 148,96 | 0,00 | 0,00 | 0,00 | 0,00 | 0,00 | 0,00 | 0,00 |
|  | Drives | 0,00 | 0,00 | 0,00 | 0,00 | 0,00 | 0,00 | 0,00 | 0,00 | 0,00 | 0,00 | 0,00 | 0,00 |
|  | PCB | 16,05 | 4 283,60 | 1,18 | 0,00 | 16 865,60 | 0,00 | 0,00 | 0,00 | 0,00 | 0,00 | 0,30 | 204,91 |
|  | Cu/Fe coils, motors | 0,10 | 23,66 | 0,35 | 276,94 | 20 412,01 | 0,00 | 0,00 | 0,00 | 0,00 | 0,88 | 0,12 | 0,10 |
|  | Display LCD | 0,00 | 0,00 | 0,00 | 0,00 | 0,00 | 0,00 | 0,00 | 0,00 | 0,00 | 0,00 | 0,00 | 0,00 |
|  | Display CRT | 6,33 | 5 998,18 | 0,58 | 0,00 | 0,00 | 0,00 | 0,00 | 0,00 | 7 136,03 | 0,00 | 0,00 | 26 655,21 |
|  | Display TFT | 0,00 | 0,00 | 0,00 | 0,00 | 0,00 | 0,00 | 0,00 | 0,00 | 0,00 | 0,00 | 0,00 | 0,00 |
|  | Al alloys | 0,00 | 15 696,20 | 0,00 | 0,00 | 0,00 | 0,00 | 0,00 | 0,00 | 401,35 | 0,00 | 0,00 | 0,00 |
|  | Cu alloys | 0,00 | 0,00 | 0,00 | 0,00 | 0,00 | 0,00 | 0,00 | 0,00 | 0,00 | 0,00 | 0,00 | 0,00 |
|  | Mg alloys | 0,00 | 0,00 | 0,00 | 0,00 | 0,00 | 0,00 | 0,00 | 0,00 | 0,00 | 0,00 | 0,00 | 0,00 |
|  | Background lighting CFL | 0,00 | 0,00 | 0,00 | 0,00 | 0,00 | 0,00 | 0,00 | 0,00 | 0,00 | 0,00 | 0,00 | 0,00 |
|  | LED | 0,00 | 0,00 | 0,00 | 0,00 | 0,00 | 0,00 | 0,00 | 0,00 | 0,00 | 0,00 | 0,00 | 0,00 |
| 0309  FPD Monitors | Cables | 0,00 | 1 259,55 | 0,00 | 0,00 | 6 195,63 | 0,00 | 0,00 | 0,00 | 0,00 | 0,00 | 0,00 | 0,00 |
|  | Drives | 0,00 | 0,00 | 0,00 | 0,00 | 0,00 | 0,00 | 0,00 | 0,00 | 0,00 | 0,00 | 0,00 | 0,00 |
|  | PCB | 17,59 | 3 693,17 | 5,73 | 5,99 | 26 227,59 | 0,00 | 2,03 | 0,40 | 0,00 | 0,36 | 0,96 | 30,69 |
|  | Cu/Fe coils, motors | 0,09 | 23,35 | 0,00 | 1,02 | 0,83 | 0,00 | 0,00 | 0,00 | 0,00 | 0,07 | 0,00 | 0,00 |
|  | Display LCD | 84,31 | 2 771,64 | 21,62 | 0,00 | 1,44 | 0,11 | 7,33 | 0,00 | 258,13 | 0,00 | 3,81 | 0,00 |
|  | Display CRT | 0,00 | 0,00 | 0,00 | 0,00 | 0,00 | 0,00 | 0,00 | 0,00 | 0,00 | 0,00 | 0,00 | 0,00 |
|  | Display TFT | 0,00 | 0,00 | 0,00 | 0,00 | 0,00 | 0,00 | 0,00 | 0,00 | 0,00 | 0,00 | 0,00 | 0,00 |
|  | Al alloys | 0,00 | 58 625,06 | 0,00 | 0,00 | 0,00 | 0,00 | 0,00 | 0,00 | 0,00 | 0,00 | 0,00 | 0,00 |
|  | Cu alloys | 0,00 | 0,00 | 0,00 | 0,00 | 0,00 | 0,00 | 0,00 | 0,00 | 0,00 | 0,00 | 0,00 | 0,00 |
|  | Mg alloys | 0,00 | 0,00 | 0,00 | 0,00 | 0,00 | 0,00 | 0,00 | 0,00 | 1 499,02 | 0,00 | 0,00 | 0,00 |
|  | Background lighting CFL | 0,00 | 0,00 | 0,00 | 0,00 | 0,00 | 0,35 | 0,00 | 0,00 | 0,00 | 0,00 | 0,00 | 0,00 |
|  | LED | 0,05 | 12,78 | 0,03 | 0,002 | 26,85 | 0,0005 | 0,01 | 0,00 | 0,00 | 0,00 | 0,003 | 0,01 |
| 0407  CRT TVs | Cables | 0,00 | 0,00 | 0,00 | 0,00 | 10 254,08 | 0,00 | 0,00 | 0,00 | 0,00 | 0,00 | 0,00 | 162,70 |
|  | Drives | 0,00 | 0,00 | 0,00 | 0,00 | 0,00 | 0,00 | 0,00 | 0,00 | 0,00 | 0,00 | 0,00 | 0,00 |
|  | PCB | 23,18 | 3 149,90 | 1,11 | 1,68 | 6 710,41 | 0,00 | 7,25 | 0,26 | 45,70 | 0,64 | 1,18 | 142,74 |
|  | Cu/Fe coils, motors | 0,04 | 8,70 | 0,00 | 9,66 | 14 031,59 | 0,00 | 0,00 | 0,00 | 0,00 | 0,32 | 0,00 | 250,50 |
|  | Display LCD | 0,00 | 0,00 | 0,00 | 0,00 | 0,00 | 0,00 | 0,00 | 0,00 | 0,00 | 0,00 | 0,00 | 0,00 |
|  | Display CRT | 7,33 | 6 950,16 | 0,67 | 0,00 | 0,00 | 0,00 | 0,00 | 0,00 | 8 268,61 | 0,00 | 0,00 | 30,89 |
|  | Display TFT | 0,00 | 0,00 | 0,00 | 0,00 | 0,00 | 0,00 | 0,00 | 0,00 | 0,00 | 0,00 | 0,00 | 0,00 |
|  | Al alloys | 0,00 | 0,00 | 0,00 | 0,00 | 0,00 | 0,00 | 0,00 | 0,00 | 0,00 | 0,00 | 0,00 | 0,00 |
|  | Cu alloys | 0,00 | 0,00 | 0,00 | 0,00 | 0,00 | 0,00 | 0,00 | 0,00 | 0,00 | 0,00 | 0,00 | 0,00 |
|  | Mg alloys | 0,00 | 0,00 | 0,00 | 0,00 | 0,00 | 0,00 | 0,00 | 0,00 | 0,00 | 0,00 | 0,00 | 0,00 |
|  | Background lighting | 0,00 | 0,00 | 0,00 | 0,00 | 0,00 | 0,00 | 0,00 | 0,00 | 0,00 | 0,00 | 0,00 | 0,00 |
|  | LED | 0,00 | 0,00 | 0,00 | 0,00 | 0,00 | 0,00 | 0,00 | 0,00 | 0,00 | 0,00 | 0,00 | 0,00 |
| 0408  FPD TVs | Cables | 0,00 | 0,00 | 0,00 | 0,00 | 1 767,85 | 0,00 | 0,00 | 0,00 | 0,00 | 0,00 | 0,00 | 0,00 |
|  | Drives | 0,00 | 0,00 | 0,00 | 0,00 | 0,00 | 0,00 | 0,00 | 0,00 | 0,00 | 0,00 | 0,00 | 0,00 |
|  | PCB | 29,24 | 10 320,32 | 2,97 | 0,75 | 14 945,65 | 0,00 | 2,73 | 0,41 | 0,00 | 0,64 | 0,69 | 28,52 |
|  | Cu/Fe coils, motors | 0,09 | 33,76 | 0,00 | 5,32 | 7,98 | 0,00 | 0,00 | 0,00 | 0,00 | 0,11 | 0,00 | 0,00 |
|  | Display LCD | 8,23 | 270,58 | 2,11 | 0,00 | 0,14 | 0,01 | 1,63 | 0,00 | 25,20 | 0,00 | 0,37 | 0,00 |
|  | Display CRT | 0,00 | 0,00 | 0,00 | 0,00 | 0,00 | 0,00 | 0,00 | 0,00 | 0,00 | 0,00 | 0,00 | 0,00 |
|  | Display TFT | 4,22 | 10 029,65 | 0,00 | 0,00 | 3,69 | 0,00 | 26,74 | 0,00 | 471,09 | 0,00 | 0,00 | 0,00 |
|  | Al alloys | 0,00 | 34 105,95 | 0,00 | 0,00 | 0,00 | 0,00 | 0,00 | 0,00 | 872,08 | 0,00 | 0,00 | 0,00 |
|  | Cu alloys | 0,00 | 0,00 | 0,00 | 0,00 | 0,00 | 0,00 | 0,00 | 0,00 | 0,00 | 0,00 | 0,00 | 0,00 |
|  | Mg alloys | 0,00 | 0,00 | 0,00 | 0,00 | 0,00 | 0,00 | 0,00 | 0,00 | 0,00 | 0,00 | 0,00 | 0,00 |
|  | Background lighting | 0,00 | 0,00 | 0,00 | 0,00 | 0,00 | 0,00 | 0,00 | 0,00 | 0,00 | 0,00 | 0,00 | 0,00 |
|  | LED | 1,45 | 380,47 | 0,84 | 0,07 | 799,05 | 0,01 | 0,42 | 0,00 | 0,00 | 0,00 | 0,10 | 0,26 |

Source: ProSUM (Huisman et al., 2017)

Table S6. Treatment scenarios by the official schemes considered in the case study in France (f-t)

| **UNU-keys** | **Scenario A** | | **Scenario B** | | **Scenario C** | |
| --- | --- | --- | --- | --- | --- | --- |
|  | **Description** | **Share**  **(%)** | **Description** | **Share**  **(%)** | **Description** | **Share**  **(%)** |
| 030301  Tablets | Shredding of the whole device via cross-flow shredder and fractions sorting | 85 | Deep-level manual dismantling of the subassemblies | 5 | Direct treatment in copper smelter after removal of the battery | 10 |
| 030302  Laptops | After removal of the battery and display panel, the entire device is treated in a medium shredder followed by fractions sorting | 50 | Manual dismantling of battery display panel and high value components. Remaining parts are forwarded to a  medium shredder followed by fractions sorting | 50 | - | - |
| 0308  0407  CRT TVs and monitors | Manual dismantling of the casings, removal of components (e.g. CRT and PCBs). Remaining parts are forwarded to a  medium shredder followed by fractions sorting | 100 | - | - | - | - |
| 0309  0408  FPD TVs and monitors | Manual dismantling of LCD screen and backlighting systems, as well as of high value components.  Remaining parts are forwarded to a  medium shredder, followed by fractions sorting | 60 | Mechanical dismantling of LCD screen, backlighting systems and PCBs greater than 10 cm^2^. Remaining parts are forwarded to a medium shredder, followed by fractions sorting | 30 | Shredding of the whole device at negative air pressure followed by fractions sorting. | 10 |

Source: Cucchiella et al., 2015; Horta Arduin et al., 2019; Huisman et al., 2008; Monier et al., 2013; Tecchio et al., 2018

Table S7 - Transfer coefficient matrix (c-f*,m-f*).

| **Fractions (f*)** | **Al** | **Cu** | **PCB** | **Plastics** | **Fe** | **Other** |
| --- | --- | --- | --- | --- | --- | --- |
| Aluminium | **88.1%** | 0.5% | 3.5% | 7.3% | 0.0% | 0.6% |
| Copper | 0.0% | **85.0%** | 0.0% | 10.0% | 0.0% | 5.0% |
| PCB | 0.2% | 0.1% | **93.1%** | 6.6% | 0.0% | 0.0% |
| Plastics | 12.1% | 2.8% | 3.4% | **77.2%** | 1.2% | 3.3% |
| Steel | 0.0% | 1.4% | 13.8% | 16.8% | **65.9%** | 2.2% |
| Other fractions | 0.0% | 6.3% | 2.9% | 90.9% | 0.0% | 0.0% |

Source: Horta Arduin et al., 2019 based on data provided by MTB Recycling.

Table S8. Efficiency of the final recycling operation per target material (f*-e).

| **Elements** |  | **Efficiency rate** |
| --- | --- | --- |
| Aluminium |  | 98% |
| Antimony (PCB) |  | 80% |
| Cobalt (PCB and batteries) |  | 90% |
| Copper |  | 70% |
| Copper (PCB) |  | 95% |
| Copper (batteries) |  | 90% |
| Silver (PCB) |  | 95% |
| Gold (PCB) |  | 97% |
| Indium (PCB) |  | 10% |
| Palladium (PCB) |  | 95% |

Source: Ardente and Mathieux, 2012; Chancerel and Marwede, 2016; Tecchio et al., 2018

**References**

Ardente, F., Mathieux, F., 2012. Integration of resource efficiency and waste management criteria in European product policies - Second phase - Report 3, Final Executive Summary. …. https://doi.org/10.2788/72577

Chancerel, P., Marwede, M., 2016. Feasibility study for setting-up reference values to support the calculation of recyclability / recoverability rates of electr(on)ic products. https://doi.org/10.2788/901715

Cucchiella, F., D’Adamo, I., Lenny Koh, S.C., Rosa, P., 2015. Recycling of WEEEs: An economic assessment of present and future e-waste streams. Renew. Sustain. Energy Rev. 51, 263–272. https://doi.org/10.1016/j.rser.2015.06.010

Horta Arduin, R., Grimaud, G., Martínez Leal, J., Pompidou, S., Charbuillet, C., Laratte, B., Alix, T., Perry, N., 2019. Influence of Scope Definition in Recycling Rate Calculation for European E-Waste Extended Producer Responsibility. Waste Manag. 84, 1–36. https://doi.org/10.1016/j.wasman.2018.12.002

Huisman, J., Leroy, P., Tertre, F., Ljunggren Söderman, M., Chancerel, P., Cassard, D., Løvik, A.N., Wäger, P., Kushnir, D., Rotter, V.S., Mählitz, P., Herreras, L., Emmerich, J., Hallberg, A., Habib, H., Wagner, M., Downes, S., 2017. Prospecting Secondary Raw Materials in the Urban Mine and mining wastes (ProSUM) - Final Report. Brussels, Belgium. https://doi.org/10.13140/RG.2.2.10451.89125

Huisman, J., Magalini, F., Kuehr, R., Maurer, C., Ogilvie, S., Poll, J., Delgado, C., Artim, E., Szlezak, J., Stevels, A., 2008. Review of Directive 2002/96 on Waste Electrical and Electronic Equipment (WEEE) - Final report. https://doi.org/07010401/2006/442493/ETU/G4

Magalini, F., Huisman, J., 2018. WEEE Recycling Economics – The shortcomings of the current business model?

Monier, V., Deprouw, A., Lecerf, L., Echinard, L., 2013. Rapport annuel sur la mise en œuvre de la réglementation relative aux Déchets d’Équipements Électriques et Électroniques (DEEE) - Rapport Annuel 2012.

Tecchio, P., Ardente, F., Marwede, M., Clemm, C., Dimitrova, G., Mathieux, F., 2018. Analysis of material efficiency aspects of personal computers product group. https://doi.org/10.2788/89220
